# Airway Mucus and Asthma: The Role of MUC5AC and MUC5B

**DOI:** 10.3390/jcm6120112

**Published:** 2017-11-29

**Authors:** Luke R. Bonser, David J. Erle

**Affiliations:** Lung Biology Center, University of California San Francisco, San Francisco, CA 94143, USA; luke.bonser@ucsf.edu

**Keywords:** MUC5AC, MUC5B, asthma

## Abstract

Asthma is characterized by mucus abnormalities. Airway epithelial hyperplasia and metaplasia result in changes in stored and secreted mucin and the production of a pathologic mucus gel. Mucus transport is impaired, culminating in mucus plugging and airway obstruction—a major cause of morbidity in asthma. The polymeric mucins MUC5AC and MUC5B are integral components of airway mucus. *MUC5AC* and *MUC5B* gene expression is altered in asthma, and recent work sheds light on their contribution to asthma pathogenesis. Herein, we review our current understanding of the role of MUC5AC and MUC5B in mucus dysfunction in asthma.

## 1. Asthma

Asthma is a common, chronic, non-communicable disease that affects ~334 million people of all ages, races, and ethnicities worldwide [1]. Dramatic increases in the prevalence of atopy and asthma have occurred in Westernized countries, and incidence is rising in less-developed countries [2]. Asthma causes approximately 250,000 deaths annually, is a major cause of lost school and work days, and imposes a substantial economic burden, particularly in low- to middle-income countries [3,4]. Asthma symptoms include wheezing, breathlessness (dyspnea), chest tightness, and cough; all result from obstruction in airflow, arising from a combination of inflammation-induced airway smooth muscle constriction and impaired mucociliary clearance [5].

## 2. Mucociliary Clearance

The conducting airways of the lung are lined by a pseudostratified columnar epithelium [6]. The epithelium is populated by several cell types. Ciliated cells are interspersed with secretory cells [6], which include club and goblet cells, and contribute secretions to the apical mucus gel [7]. In larger airways, the surface epithelium is contiguous with submucosal glands, which are situated between smooth muscle and cartilage plates [7]. Mucous cells within gland acini are a major mucus source [7] and mucous cells are also found within ducts which deliver gland secretions to the airway lumen. Basal cells anchor the epithelium to the underlying matrix and function as stem/progenitor cells for other airway cell types during natural turnover and in response to injury [8].

Together, the ciliated epithelium, periciliary layer, and airway mucus gel form the mucociliary escalator [9]. Individual cilia atop ciliated cells beat in concert within the periciliary layer to propel airway mucus up and out of the lung [10]. In addition to providing a favorable environment for ciliary activity, the periciliary layer prevents compression from the overlying mucus gel layer, and provides a water reservoir to control water distribution [11]. Airway mucus is a hydrogel that functions as molecular flypaper, protecting the underlying epithelium by trapping potentially harmful inhaled particles, pathogens, and dissolved chemicals within it [12].

Effective mucociliary clearance is essential for maintaining an uninfected and unobstructed airway, and relies on ciliary activity and the physiochemical properties of the periciliary layer and mucus gel [9,12]. Failure of any component of the mucociliary apparatus can render clearance defective and lead to obstruction. For example, in primary ciliary dyskinesia (PCD), cilia absence and/or immotility impair mucociliary clearance, while in cystic fibrosis (CF), periciliary liquid depletion manifests as mucostasis [11]. In asthma, ‘pathologically the outstanding feature of the asthmatic lung lies in the failure of clearance of the bronchial secretions’ [13]. In fact, the principal cause of death in asthma is asphyxiation from intraluminal airway obstruction by mucus plugs [14,15,16]. Defective mucociliary clearance is observed even in mild stable asthma [17,18,19] and clearance decreases further during acute exacerbation [20].

## 3. Polymeric Mucins

Mucins are the products of secretory cells and the primary macromolecular components of mucus. Mucins are heterogeneous, densely glycosylated high-molecular-weight molecules [21]. To date, ~20 mucin-like genes have been identified and fall into 2 broad classes: membrane-bound (or cell surface) mucins and secreted mucins. Secreted mucins are further subdivided into polymeric and non-polymeric glycoconjugates [22]. Four polymeric mucin genes, *MUC2*, *MUC5AC*, *MUC5B*, and *MUC6*, are present in tandem on a conserved cluster of human chromosome 11p15 and likely arose by gene duplication [23]; the fifth, *MUC19*, is found on 12q12 [21,24].

**Figure 1:**
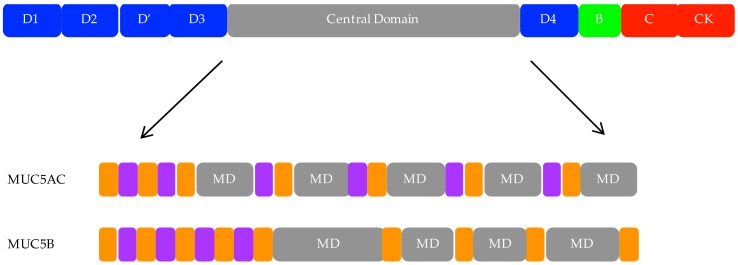
Multidomain structure of the mucins 5AC and 5B (MUC5AC and MUC5B). Top: generic representation of a polymeric mucin. Secreted polymeric mucins possess cysteine-rich vWf-like amino (D1, D2, D’, and D3) and carboxy (D4, B, C, and CK) terminal domains, important for dimerization and polymerization of polymeric mucins, as well as a large, central domain. Bottom: Central domain organization of MUC5AC and MUC5B. The hallmark of the MUC family members are the mucin domains (MDs). These domains are unique in sequence and size, and vary in length and number between different mucins including MUC5AC and MUC5B. They are rich in serine, threonine, and proline, and are the site of *O*-glycosylation. Additionally, the central region contains repetitive (grey), non-repetitive (purple), and cysteine-rich (orange) domains.

Polymeric mucin gene products have complex, multidomain polypeptide structures important to their function (Figure 1) [21]. They possess cysteine-rich von Willebrand factor (vWf)-like D-domains including 3 D-domains (D1, 2, and 3) at the N terminus and a fourth at the C terminus (D4); a partial D domain (D’) lies between the D2 and D3 domains [21,25]. Additional cysteine-rich vWf-like domains (B, C, CK) are located at the C terminus. These domains are sites of mucin dimerization and polymerization, forming disulfide-bonded polymers polydisperse in both mass (2–50 mDa) and length (0.5–10 μm) [9]. The capacity of polymeric mucins to polymerize is crucial to their gel-forming properties.

The hallmark of these proteins is the tandem repeat or mucin domains, encoded by a single large central exon and rich in proline, serine, and threonine residues [9]. These regions are the site of *O*-glycosylation; the repetitive sequences create a dense array of glycan structures which contribute 50–90% weight by mass of the glycoprotein [21]. The extensive glycosylation extends and stiffens the mucin polypeptide chain. Terminal sulfation and sialyation of the *O*-glycans results in charge repulsion between neighboring oligosaccharide groups. Mucins therefore have a large hydrodynamic volume in solution, which is important for gel formation [21]. Charged polymers, like mucins, are also very effective lubricants in aqueous environments [26]. The mucin domains are interspersed by internal cysteine-rich regions termed cys domains [21].

As aforementioned, mucins share sequence similarity with vWF, which also polymerizes through N- and C-terminal disulfide linkages [25]. Studies on intact mucins and recombinant N- and C-terminal domains, as well as studies on porcine submaxillary mucin (PSM), have shown that polymeric mucins also share some basic pattern of polymer assembly with vWF [27,28,29,30]. The latter stages of assembly involving multimerization and packaging into secretory granules are less clearly resolved. Polymeric mucins are packaged highly condensed and dehydrated into secretory granules; calcium ions enable this through shielding charge on the polyanionic mucins [31,32]. A recent study characterized an additional non-covalent association between MUC5B N-terminal D3 domains that enables secretory granule storage: uncoupling of the D3-mediated results in expansion during exocytosis [33]. As all polymeric mucins share sequence identity, it is possible that the assembly mechanism is also shared.

Additionally, the mechanisms leading to mucus formation post-exocytosis are poorly understood. A two-phase model has been proposed to explain the rapid and massive mucin expansion that occurs on secretion [34]. Following secretory granule fusion with the plasma membrane, calcium ions are exchanged by monovalent cations such as sodium and potassium and/or sequestered by bicarbonate [35,36]. This exposes the negatively charged terminal sugars on adjacent mucins, leading to their mutual repulsion and further expansion [36]. This process is followed by changes in mucin morphology, the molecules unfurling to attain a linear polymeric form in a process referred to as ‘maturation’ [34].

## 4. MUC5AC and MUC5B

Mucin production in the normal human proximal airway (defined as airways supported by cartilage and containing submucosal glands) has been explored using various techniques. Mucin gene expression (mRNA) has been demonstrated using in situ hybridization and Northern blotting. Stored mucin protein has been evidenced with general carbohydrate stains (e.g., Alcian blue–Periodic Acid Schiff stain) and more specifically by immunostaining with mucin-specific antisera. Antibody-based techniques—and, more recently, mass spectrometry—have also been used to evaluate mucin protein in airway secretions. Multiple northern blot, reverse transcription-polymerase chain reaction (RT-PCR), and in situ hybridization studies indicate that *MUC5AC* and *MUC5B* mRNA are readily detected in human airways. *MUC5AC* transcripts are restricted to goblet cells within the tracheal and bronchial epithelium [37,38,39,40]. *MUC5B* mRNA is localized to the submucosal gland and submucosal gland duct epithelium—and, to a lesser extent goblet cells—in both the tracheal and bronchiolar epithelium [37,38,39,40]. Most studies have looked at mucin expression in proximal airways, but both MUC5AC and MUC5B mRNA have also been evidenced in distal airways (defined as airways lacking cartilage and submucosal glands, and <2 mm diameter) [40]. *MUC6* was not detected in the normal human adult proximal airway, and levels of *MUC2* and *MUC19* expression are reportedly quite low [21,41].

At the protein level, biochemical analyses of respiratory secretions revealed the presence of 3 major protein species: MUC5AC and 2 glycoforms of MUC5B, termed high- and low-charge due to differing levels of sulfation [42,43,44,45]. MUC2 is a minor component of airway secretions as determined using antibodies and mass spectrometry, and we will focus on MUC5AC and MUC5B [44,46]. Immunohistochemistry has been used to identify their cellular origins and is in agreement with in situ analysis that MUC5AC and MUC5B production is spatially separated. MUC5B protein is localized to mucous cells in submucosal glands and, to a lesser extent, secretory cells within the surface airway epithelium of the trachea and bronchi [43,47,48]. The high-charge MUC5B variant has been identified in a subpopulation of submucosal gland cells indicating a distinct cellular origin and glycosyltransferase repertoire [43]. MUC5AC is localized to goblet cells in the surface epithelium and in the terminal secretory ducts of submucosal glands, but not within the gland acini [47,48,49]. In a study of the normal distal epithelium, the majority of airways stained for MUC5B [50]. A subpopulation of these airways also stained for MUC5AC, but no airways stained exclusively for MUC5AC and not MUC5B [51]. In both proximal and distal airways, MUC5AC and MUC5B are produced by different cells, or from different granules within the same cell, and remain largely segregated after secretion into the lumen (immunostaining) [50,52,53,54]. Extracellularly, MUC5AC and MUC5B may also form distinct morphologic structures: staining with lectins preferentially recognizing each mucin suggests that MUC5B forms strands and MUC5AC forms threads and sheets in a porcine model, and that MUC5AC may coat submucosal gland MUC5B bundles [54,55].

As the major matrix-forming macromolecules in airway mucus, the viscoelastic properties of airway mucus depend on MUC5AC and MUC5B [9]. Electron microscopy revealed that MUC5AC and MUC5B polymers are long, flexible linear threads [56,57]. However, MUC5AC and MUC5B differ in charge and shape [58]. Differences in MUC5AC and MUC5B result from differential glycosylation: in mice, MUC5AC is heavily fucosylated, whereas MUC5B is primarily sialylated [58]. In humans, MUC5B exists as 2 glycoforms, differing in charge due to glycosylation (sulfation) [43,45]. MUC5AC has a lower sedimentation rate than MUC5B. As both form polymers of similar size, the difference in sedimentation is likely determined by the shape of the molecules: MUC5AC behaves more rod-like or extended in solution compared with MUC5B [57]. This characteristic of MUC5AC likely explains why MUC5AC polymers appear less polydisperse than MUC5B polymers, since the extended structure gives poorer separation by sedimentation rate [43,57]. However, it must be noted that these studies were performed on mucins isolated using highly chaotropic agents (6–8 M guanidinium chloride) and analyzed in their non-native state.

Targeting mouse mucin genes has provided insights into the roles of MUC5AC and MUC5B in the airway. In wild-type mice *Muc5b* mRNA is the dominant gel-forming mucin expressed (40-fold higher than *Muc5ac*), and is also the major glycoprotein although it is not always detected by staining due to constitutive secretion [59]. Murine MUC5B is critical for mucociliary clearance and airway defense [60]. *Muc5b*-deficient mice accumulate aspirated materials in the airway and develop chronic bacterial infections, severe inflammation, and airway obstruction. Loss of MUC5B also inhibits innate inflammatory responses suppressing interleukin-23 (IL-23) and resulting in accumulation of alveolar macrophages with impaired ability to phagocytose and clear *Staphylococcus aureus* (*S. aureus*) [60]. MUC5B-overexpressing (*Scgb1a1-Muc5b*) transgenic mice have normal mucociliary transport and anti-bacterial defense and increased IL-23 production, macrophage activation, and *S. aureus* elimination [60]. The role of MUC5B was also explored in a model of CF: *Scnn1b*-Tg mice, which exhibit mucus hyperconcentration and airway surface adhesion due to overexpression of the epithelial sodium channel (ENAC), were crossed with *Muc5b*-deficient mice [61]. The magnitude of mucus obstruction in *Scnn1b*-Tg mice was significantly reduced in the absence of MUC5B; however, mucus adhesion persisted and *Muc5b* deletion did not alleviate bacterial burden [61]. Absence of MUC5B in *Scnn1b*-Tg mice was also associated with increased airway inflammation, suggesting that MUC5B is required to maintain immune homeostasis and is important in anti-bacterial defense [61].

MUC5AC-deficient mice have normal mucociliary transport and anti-bacterial defense [60]. However, a role for MUC5AC in asthma pathogenesis was established using models of allergic asthma (ovalbumin sensitization and challenge and exposure to *Aspergillus oryzae* extract (AOE)) [62]. Wild-type mice challenged with either ovalbumin or AOE exhibit significant airway hyperreactivity (AHR) in response to methacholine; however, in *Muc5ac* knockout mice, AHR was abolished following allergen challenge [62]. The authors proceeded to show that the severity and abundance of mucus plugging was significantly reduced in MUC5AC-deficient mice compared with wild-type mice following allergen challenge [62]. They concluded that MUC5AC secretion, in addition to airway smooth muscle contraction, is necessary for AHR [60,62]. Overexpression of *Muc5ac* confers resistance to viral infection but does not cause metaplasia or obstruction, suggesting mucus hypersecretion alone is insufficient to trigger plugging [63]. However, MUC5AC appears to be detrimental in acute lung injury, enhancing neutrophil trafficking and inflammation [64].

Whether the polymeric mucins function similarly in humans has yet to be established. As aforementioned, the airways of normal mice more resemble human distal airways with respect to their diameter [65]. Additionally, the distribution of secretory cells differs between human and mice; submucosal glands are limited to the laryngeal region of trachea in mice [66]. Based on these cross-species anatomical differences, one could hypothesize that MUC5B may perform baseline barrier and clearance functions in human distal airways; in support of this, *MUC5B* overexpression and protein accumulation was observed in the distal lungs in idiopathic pulmonary fibrosis, indicating impaired clearance [50,51]. In proximal airways, however, MUC5B function may be augmented by MUC5AC, since MUC5AC production is greater than in distal airways [49]. Notably, the proportion of MUC5AC and MUC5B varies with the state of health, and the effects of this in asthma are discussed below.

## 5. Regulation of *MUC5AC* and *MUC5B* Expression in Asthma

Variants in the 11p15 *MUC5B* and *MUC5AC* locus have been associated with AHR in asthma (6). Many individuals with asthma have increased *MUC5AC* mRNA levels but decreased *MUC5B* mRNA levels [67]. *MUC5AC* and *MUC5B* expression is sensitive to a wide variety of stimulants and developmental cues. Of particular pertinence to asthma are type 2 immune cells, including type 2 T helper (Th2) cells and innate lymphoid cells (iLC2s), which orchestrate allergic airway remodeling in asthma. IL-13 is produced by these cells during allergic inflammation, inducing characteristic changes in airway epithelial mRNA [68,69,70] and miRNA expression [71] in airway epithelial cells. The IL-13 transcriptional signature can be used to identify individuals with type 2 high and type 2 low asthma; approximately 50% of people with asthma are type 2 high and exhibit worsened AHR to methacholine, higher serum IgE, and eosinophils [67,69]. Individuals with type 2 high asthma also have elevated levels of *MUC5AC* compared with healthy controls or individuals with type 2 low asthma; a substantial decrease in *MUC5B* expression is also observed in type 2 high asthma [67,72]. IL-13 significantly and consistently increases expression of *MUC5AC* in human airway epithelial cells in vitro and expression of *Muc5ac* in murine models [52,68,70,73,74]. The effect of IL-13 on MUC5B is more variable. IL-13 (and allergen challenge) induces *Muc5b* in mouse models, but IL-13 frequently decreases *MUC5B* in cultured human airway epithelial cells and may reflect inter-species differences [52,72].

The link between type 2 inflammation and airway structural cell dysfunction is incompletely understood. A recent study has suggested that type 2 inflammation is necessary but not sufficient for allergic asthma, and that the airway epithelium is more responsive to type 2 inflammation in people with asthma as measured by MUC5AC [75]. Whether this is because asthma cells are intrinsically more sensitive to type 2 inflammation or develop altered responses in a chronic inflammation environment remains undetermined [75].

Epidermal growth factor receptor (EGFR) signaling is also required for mucus production in vitro and in vivo [70,76,77]. EGFR levels are increased in individuals with asthma and expression correlates with disease severity [78]. Various stimuli (bacterial products, viruses, cigarette smoke, and inflammatory cell products) and various ligands (EGF, TGF-α, amphiregulin) can trigger EGFR signaling in airway epithelial cells. EGFR signaling induces *MUC5AC* expression while EGFR tyrosine kinase inhibition blocks *MUC5AC* expression [76].

Recently, a murine inbred strain study revealed that a large fraction of the variation in secreted MUC5AC and MUC5B was attributable to strain-specific genetic differences, indicating heritability [79]. Although *MUC5AC* and *MUC5B* mRNA levels were strongly correlated, likely due to shared transcriptional regulation, neither mRNA correlated with protein production, suggesting that post-transcriptional events were important in mucin regulation [79]. Quantitative trait locus (QTL) mapping identified distinct, *trans* protein QTL for *MUC5AC* (chromosome 13) and *MUC5B* (chromosome 2) explaining 18% and 20% of phenotypic variance, respectively, indicating separate distal regulatory control [79]. Identifying additional QTL loci will inform mucin regulation further.

## 6. Goblet Cell Fate in Asthma

A key feature of airway epithelial remodeling in asthma is increased goblet cell number, which accompanies the aforementioned increase in *MUC5AC* copy number. In fatal asthma, a 30-fold increase in goblet cell number was reported [80]; increased goblet cell number is readily observed in mild to moderate disease, too [81]. The mechanisms mediating increases in goblet cells are incompletely understood with both hyperplasia and metaplasia proposed. In the human airways, understanding of secretory cell fate is limited and much of our knowledge derives from mouse models. During normal development, epithelial cells are thought to differentiate into ciliated and secretory lineages from basal and club cells that are considered to function as progenitor/stem cells. In the proximal airways, basal cells are the progenitors of ciliated and secretory cells [8]. The proportion of basal cells in the airway is highest in the large airways and progressively decreases down the tracheobronchial tree, where club cells likely act as progenitors [82].

Pathologic remodeling is caused by dysregulation of signaling cascades that govern normal differentiation. Notch signaling is an evolutionary conserved pathway that regulates cell fate decisions during development. Notch recently emerged as a pivotal regulator of basal cell differentiation in conducting airways with activation of secretory over ciliated lineages [83]. Notch2 is a common node downstream of IL-13 and is absolutely required for goblet cell metaplasia in vitro and in vivo [84]. Inhibition of Notch2 inhibits IL-13 and allergen-driven goblet cell metaplasia in vivo [84]. In a mouse model of respiratory disease, inhibition of JAG, a ligand for the transmembrane Notch receptor, reduced goblet cell metaplasia when administered prior to an inflammatory stimulus, and reversed goblet cell metaplasia when administered post-stimulus (i.e., once metaplasia was established) [85].

At the transcriptional level, a number of transcription factors are thought to be involved in increased *MUC5AC* expression and mucous metaplasia. SAM pointed domain-containing ETS transcription factor (SPDEF) is sufficient and necessary for goblet cell metaplasia and for increasing *MUC5AC* and *MUC5B* expression [86]. *SPDEF* expression is increased in airway epithelial cells of patients with asthma compared with healthy controls [87], remains increased in spite of anti-inflammatory treatment, and is upregulated following IL-13 stimulation [88]. *SPDEF* induction following IL-13 stimulation was accompanied by DNA hypomethylation of several CpG sites within the *SPDEF* promoter [89]. Epigenetic editing of *SPDEF* suppressed *MUC5AC* expression in human airway epithelial cells [90]. In *SPDEF*-deficient mice, goblet cells are absent, whilst overexpression of *SPDEF* causes goblet cell metaplasia [91].

**Figure 2:**
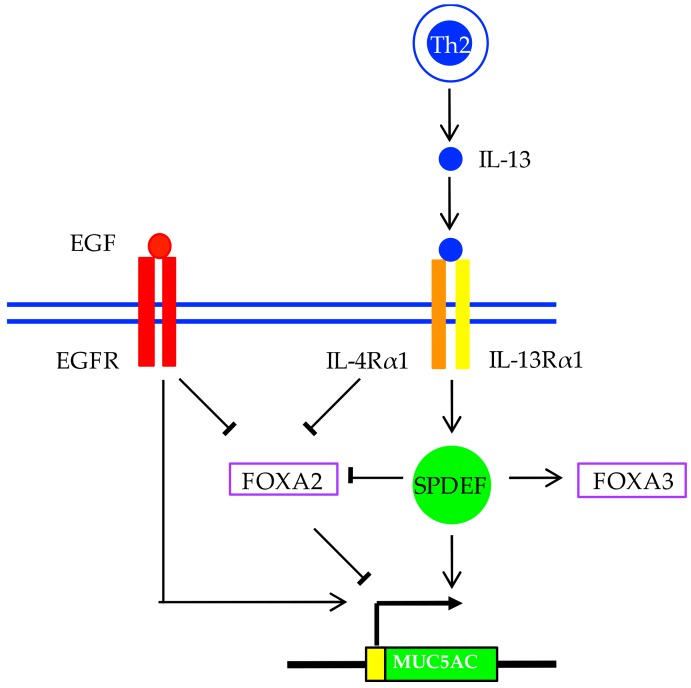
*MUC5AC* induction and goblet cell differentiation in asthma. Goblet cell metaplasia and hyperplasia are induced by various inflammatory mediators, including type 2 cytokines (e.g., interleukin-13 (IL-13)), and after activation of epidermal growth factor receptor (EGFR) as well as Notch signaling (not illustrated). Goblet cell differentiation is dictated by a large network of regulators, in which transcription factors including SAM pointed domain-containing ETS transcription factor (SPDEF) and the forkhead transcription factor family members, Forkhead box protein A2 (FOXA2) and Forkhead box protein A3 (FOXA3), interact. SPDEF is sufficient and necessary for goblet cell metaplasia, which is regulated in part by FOXA2 inhibition and FOXA3 induction. These pathways are required for MUC5AC induction and goblet cell differentiation.

Several forkhead box family members have also been implicated in airway polymeric mucin expression and mucous metaplasia. Forkhead box protein A2 (FOXA2) inhibits *SPDEF* and *MUC5AC* expression, and is a potent inhibitor of goblet cell differentiation [91,92,93]. *FOXA2* is also regulated by DNA methylation [89]. Interestingly, both IL-13 and EGFR signaling cascades converge on FOXA2 inhibition, perhaps representing a common pathway for IL-13 and EGFR-induced mucous metaplasia [10]. Another family member, *FOXA3*, also functions as a goblet cell metaplasia regulator: it is highly expressed in patients with asthma, and is IL-13- and rhinovirus-inducible [87]. HIF-1 is also downstream of IL-13 and EGF stimulation, plus an HIF-1 binding motif is conserved in mammalian *MUC5AC* promoters [94]. *MUC5AC* induction in asthma is summarized in Figure 2.

## 7. Altered MUC5AC and MUC5B Properties

Abnormalities in goblet cell number are accompanied by changes in stored and secreted mucin. In asthma as in health, MUC5AC is produced in goblet cells from the surface epithelium, while MUC5B is largely produced in the submucosal glands. However, changes in the relative proportion of MUC5AC and MUC5B are observed in asthma (Figure 1). As at the gene expression level, elevated MUC5AC production is consistently reported, but there are conflicting reports regarding MUC5B. Increased MUC5AC and MUC5B protein have been reported in sputum from individuals with asthma [44]. Another study identified MUC5B as the predominant mucin in healthy secretions, while MUC5AC concentration increased and MUC5B decreased in individuals with asthma, including those in exacerbation [53]. Interestingly, within the asthma cohort, a higher ratio of MUC5AC to MUC5B correlated with type 2 inflammation (>2% eosinophils) [53]. In pediatric asthma, similar observations were made: increased MUC5AC was reported in children with both stable and acute asthma versus healthy controls. Median MUC5B concentration was non-significantly reduced; however, overrepresentation of a low-charge MUC5B glycoform was observed [95]. A study on viscous mucus exudate from a patient who died in *status asthmaticus* demonstrated that the MUC5B low-charge glycoform was the major constituent [96]. The *O*-secretor mucin glycan phenotype (addition of a terminal α1,2-fucosylation by FUT2 at epithelial surfaces) is associated with severe asthma exacerbation risk, and MUC5AC is heavily fucosylated [58,97]. These data suggest mucin glycosylation status is important in asthma and may modify gel-forming capabilities of mucins and/or interactions with non-mucin host-defense molecules or pathogens. Additionally, in mild asthmatics, large amounts of glandular MUC5B extracellular mucus was observed [47]. The contribution of glandular MUC5B to mucus dysfunction in asthma requires further exploration.

It has been noted that asthmatic sputum is abnormally viscous [98]. Rheological measurements of sputum from patients with asthma during acute exacerbation demonstrated increased elastic and viscous moduli; the increased elastic response dominated, suggesting increased crosslinking of mucin polymers as demonstrated recently in CF [99,100]. These differences are most visible in the form of mucus plugs, which occlude airways and prevent mucociliary clearance. Although mucins are primarily responsible for the biophysical properties of the gel, other constituents including DNA and albumin may also contribute to the increased viscoelasticity reported in asthma [98,99]. Several studies have compared the size distribution of MUC5AC and MUC5B in sputum from patients with asthma, yet no discernible difference has been observed [95,99]. It has been suggested that mucin degradation is inhibited in asthma: protease-dependent mucin degradation was inhibited at the height of exacerbation but restored during recovery [99]. Alterations in protease and antiprotease expression has been reported in asthma, suggesting that imbalance could affect mucus clearance and contribute to tethering and plugging [69,101].

**Figure 3:**
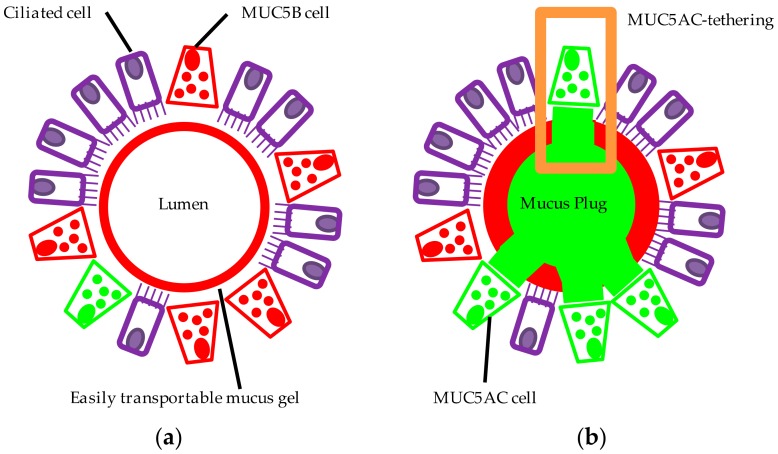
Alterations in MUC5AC and MUC5B contribute to mucus dysfunction in asthma. In this simplified model of the proximal healthy airway (**a**), MUC5B (red) is the predominant mucin produced and the principal component of the airway mucus gel (the secretion originates in part from submucosal glands not shown). The MUC5B-rich gel is readily transported by the ciliated epithelium (purple) maintaining an unobstructed and uninfected airway. In asthma (**b**), mucin expression is altered: MUC5AC (green) production is upregulated, while MUC5B production is reduced. This culminates in a heterogeneous airway mucus gel comprising distinct MUC5AC and MUC5B domains. Extracellular MUC5AC domains remain tethered to MUC5AC-producing cells (orange box) compromising mucociliary clearance. Mucus accumulates, forming mucus plugs which occlude the airway. Airway obstruction manifests clinically as breathlessness and wheeze; in some patients, intraluminal occlusion by mucus plugging can lead to asphyxiation.

Despite substantial plugging observed in the majority of patients with fatal asthma, the biochemical and biophysical mechanisms by which secreted mucus occludes airways is not fully understood. An autopsy study demonstrated a large increase in the frequency of goblet cells in continuity with intraluminal mucus in individuals with asthma [102]. We found that extracellular domains of MUC5AC-rich mucus were intimately associated with or tethered to epithelial mucous cells, markedly impairing mucociliary transport [52]. Images from allergic mouse airways [62] are also consistent with tethering. MUC5AC-tethering probably leads to progressive luminal accumulation of mucus and airway plugging (Figure 3). The mechanism by which MUC5AC is tethered requires further investigation. It is possible that exocytosed MUC5AC is not fully released or expanded from goblet cells, leading to tethering of mucus to the epithelium.

Interestingly, mucins from a viscous mucus plug appeared as ‘tangled masses condensed around nodes from which many chains emanate’, contrasting with the classic view of polymeric mucins as linear threadlike molecules [96]. These mucins resembled freshly secreted mucins, implicating improper unpackaging of mucins post-secretion as a contributor to tethering [34]. Defective postsecretory maturation of MUC5B has been reported in CF, where the absence of functional CFTR results in a volume-depleted airway surface, reduced bicarbonate ion concentration, and a lower pH [103]. Lower pH influences airway surface liquid (ASL) viscosity and likely inhibits MUC5B unpackaging through impaired calcium chelation by bicarbonate and/or by altering the proteolytic environment [103,104]. Altered luminal pH could also contribute to asthma since sputum samples collected during asthma exacerbation have been shown to be more acidic compared with samples from people with stable asthma or with other respiratory diseases [102,105]. As aforementioned, a plethora of transcriptional alterations are observed in asthma which likely alter ciliary transport and ion transport and could impact airway surface pH, mucus adhesivity, and mucociliary transport. The implications of altered pH on mucociliary transport are adequately demonstrated in cystic fibrosis [11].

We also demonstrated that MUC5AC and MUC5B form distinct extracellular domains and that IL-13 induces a heterogeneous gel, which could have implications for mucociliary clearance in asthma [52]. Differences in the biophysical properties of extracellular MUC5AC- and MUC5B-containing domains could be attributable to intrinsic properties of the MUC5AC and MUC5B core proteins. Other potentially important factors include post-translational mucin modifications including glycosylation (low-charge MUC5B [95]) or disulfide cross-linking, hydration- and bicarbonate-mediated mucin expansion, non-mucin mucus constituents (e.g., intelectin-1 [106]), or other differences in the secretory cell or the luminal environment. Additionally, differences in airway mucus may be caused by differences in the clinical features of asthma, including exacerbation, severity, duration, smoking, and treatment.

## 8. Therapeutics

Together with smooth muscle contraction, mucus accumulation is a major cause of airway obstruction in asthma [10]. A mucoactive drug is an agent capable of modifying mucus production, secretion, properties, or its interactions with the mucociliary epithelium [107]. Mucoactive agents facilitate mucus clearance (mucolytics) or inhibit mucus production or secretion (mucoregulators) [108,109].

No effective mucolytic treatments for asthma exist. Hypertonic saline is associated with mucus clearance but modest improvements in airflow [110]. In a recent study, treating acidic ASL with hypertonic saline reduced viscosity, suggesting that acidic pH influences mucin electrostatic interactions [104]. The reducing agent N-acetylcysteine (NAC) is sometimes used as a mucolytic therapy but exhibits low efficacy (low mucolytic activity at high mucus concentrations and neutral pH) and tolerability: N-acetylcysteine can irritate the airways and cause bronchospasm in hyperreactive patients [111,112,113,114,115,116]. Thiol-modified carbohydrates have been proposed as novel mucolytics for CF and other lung diseases [100]. Pulmozyme, a human DNAse, improves pulmonary function and reduces pulmonary exacerbation in CF [117,118], but there is no evidence it is effective in other diseases, presumably since it does not target the mucins themselves. Recent evidence suggests mucin-specific approaches could be of benefit: complete MUC5B removal from the airway may be detrimental [61], whilst the identification of MUC5AC as an essential non-contractile mediator of AHR [62] and the role of MUC5AC in tethering [52] suggest MUC5AC-specific therapies could be of benefit in asthma.

Inhaled corticosteroid (ICS) and ß-adrenergic agonist therapy are mainstays of asthma and are effective in reducing symptoms and exacerbation frequency and improving pulmonary function [119,120]. In animal models, ICS use is associated with a reduction in goblet cell volume [121,122,123]. A recent study demonstrated that ICS treatment of human bronchial epithelial cells inhibited IL-13–induced goblet cell metaplasia, decreasing the number of MUC5AC-positive goblet cells and ATP-stimulated mucus secretion [72]. Corticosteroid treatment also restored MUC5B protein levels [72]. The addition of a long-acting ß-adrenergic agonist modestly augmented these effects [72]. These data indicate that commonly used asthma therapies may have mucoregulatory roles. Omalizumab, a monoclonal antibody directed against IgE, was the first biologic approved for asthma, and has been shown to reduce the number of exacerbations and improve symptom severity, although the role of omalizumab as a mucoregulator has not been established [124].

There is rationale for development of further biologics and/or cytokine-specific antibodies as mucoregulators. For example, a recent trial with anti-IL-13 (lebrikizumab) demonstrated improved airflow (prebronchodilator FEV1) in patients with type 2 high asthma [125]. However anti-IL-13 only met its primary endpoint of significantly reducing exacerbation rates in one of two parallel Phase III studies, despite significantly improving FEV1 in both [125,126]. Although other mechanisms likely contribute, improved FEV1 could represent reduced mucus obstruction. An agent (dupilumab) that inhibits a common subunit of IL-13 and IL-4 receptors also had beneficial effects in a clinical trial [127]. This, however, is not a comprehensive list, and there are other biologics that may have mucoregulatory roles also in development. Agents that reduce production of IL-13, including antibodies against the epithelial-derived type 2-promoting cytokine TSLP likely act in part by inhibiting the production of pathologic mucins [72,128].

Other approaches to regulating mucus obstruction might include specific targeting of mucin (especially *MUC5AC*) gene transcription or protein processing, or blocking the differentiation of mucus-producing goblet cells. For example, targeting Notch through Jagged antagonism reversed goblet cell metaplasia in a preclinical model [85]. Although controlling mucus hypersecretion is attractive therapeutically, a therapy which completely disrupts secretion and inhibits MUC5B may be detrimental. Overall, it is unclear which mucoactive drugs would offer optimal benefit [129] and further research is required.

## 9. Conclusions

MUC5AC and MUC5B are principal components of airway mucus. In the airway, their production is spatially separated, and they serve different functions (at least in mice). Altered MUC5AC and MUC5B gene expression is consistently observed in both asthma models and individuals with disease. Increases in goblet cell number accompany changes in mucin gene expression, which result in altered mucus composition and organization. These changes are associated with increased gel viscoelasticity, and are sufficient to impair mucus transport through MUC5AC-tethering, likely contributing to airway obstruction and mucus plugging. Continued research to understand the mechanisms underlying goblet cell fate and MUC5AC-tethering are crucial for the development of effective mucoactive agents.
